# The Plastered Skulls from the Pre-Pottery Neolithic B Site of Yiftahel (Israel) – A Computed Tomography-Based Analysis

**DOI:** 10.1371/journal.pone.0089242

**Published:** 2014-02-19

**Authors:** Viviane Slon, Rachel Sarig, Israel Hershkovitz, Hamoudi Khalaily, Ianir Milevski

**Affiliations:** 1 Department of Anatomy and Anthropology, Sackler School of Medicine, Tel Aviv University, Tel Aviv, Israel; 2 Department of Orthodontics, Maurice and Gabriela Goldschleger School of Dental Medicine, Tel Aviv University, Tel Aviv, Israel; 3 Department of Excavations, Surveys and Research, Israel Antiquities Authority, Jerusalem, Israel; University of Kansas, United States of America

## Abstract

Three plastered skulls, dating to the Pre-Pottery Neolithic B, were found at the site of Yiftahel, in the Lower Galilee (Israel). The skulls underwent refitting and restoration processes, details of which are described herein. All three belong to adults, of which two appear to be males and one appears to be a female. Virtual cross-sections were studied and a density analysis of the plaster was performed using computed tomography scans. These were utilized to yield information regarding the modeling process. Similarities and differences between the Yiftahel and other plastered skulls from the Levant are examined. The possible role of skull plastering within a society undergoing a shift from a hunting-gathering way of life to a food producing strategy is discussed.

## Introduction

**Figure 1:**
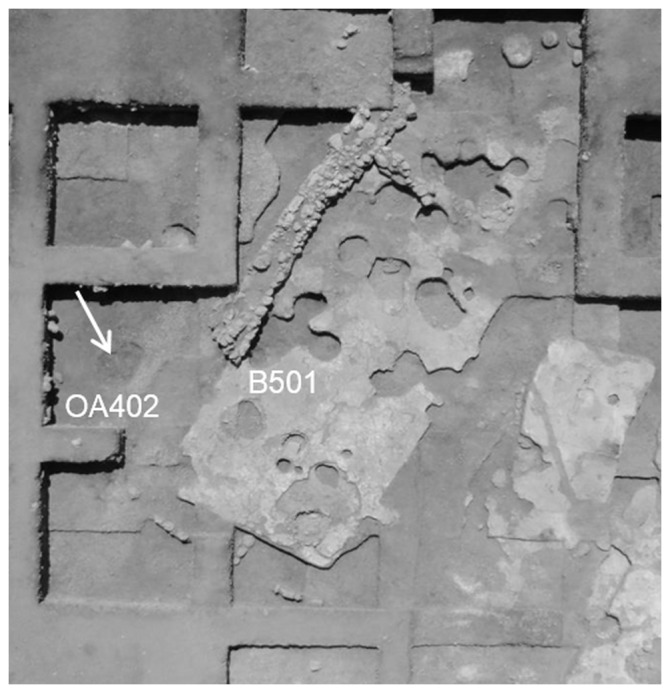
Aerial photograph of Area I in the site of Yiftahel. Location of the three plastered skulls is marked by a white arrow (OA – open area; B – building). Many of the disruptions in the plastered floor of this building were related to human burials.

**Figure 2:**
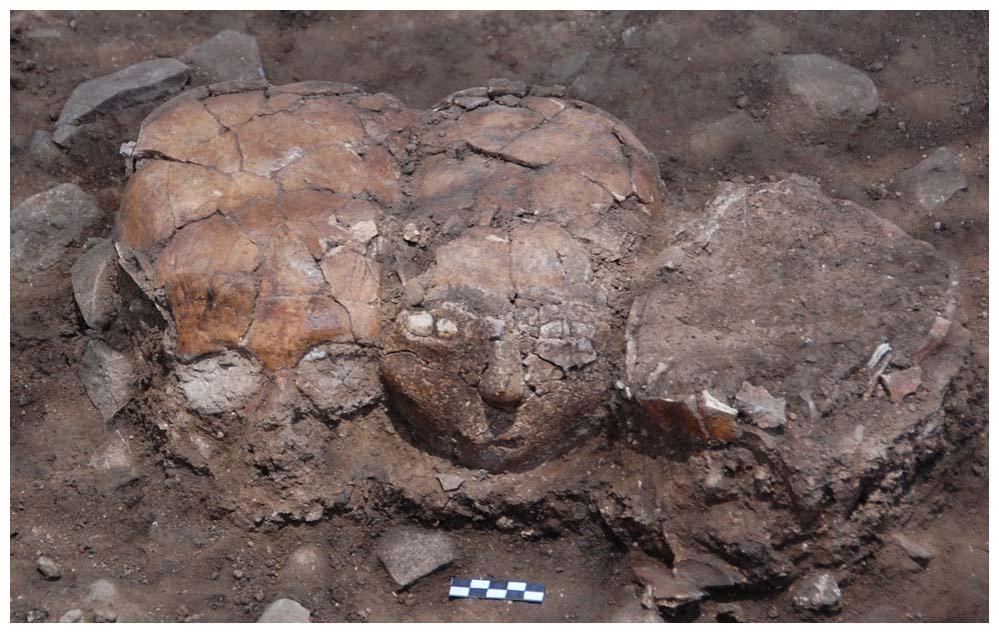
The three plastered skulls *in situ*. The plastered skulls are facing west (L4187, open area 402).

Yiftahel (Khalet Kalladyiah) is located in the Lower Galilee (Israel) (map ref. NIG 221656–2006/739873–40307), ca. 5 km west of Nazareth. Several excavations were conducted at the site since the 1980s (see summary in [1]). Some of the present authors conducted two seasons of excavations in 2007 and 2008, finding remains dating to the Pre-Pottery Neolithic B (PPNB), the Pottery Neolithic (PN) (Lodian and Wadi Rabah cultures) and the Early Bronze Age. During the 2008 excavation, three plastered skulls were uncovered in Area I, in the northern section of the site (Figs. 1,2), outside the living quarters, and dated to the mid-late PPNB [2], [3]. The skulls were found in Stratum 3 d, a phase of the site which belongs to the PPNB and was roughly dated by ^14^C to ca. 9000–8500 uncalibrated B.P. using short-lived samples (horsebeans and lentils) from previous excavations at the site [1] and using samples from the 2007–2008 excavations (E. Boaretto, personal communication). Some of the ^14^C dates provided from plaster floors in Area I fall within these chronological ranges. Deviation from these dates based on other plaster floor samples may be due to the methods used to extract charcoal from the lime [4].

**Figure 3:**
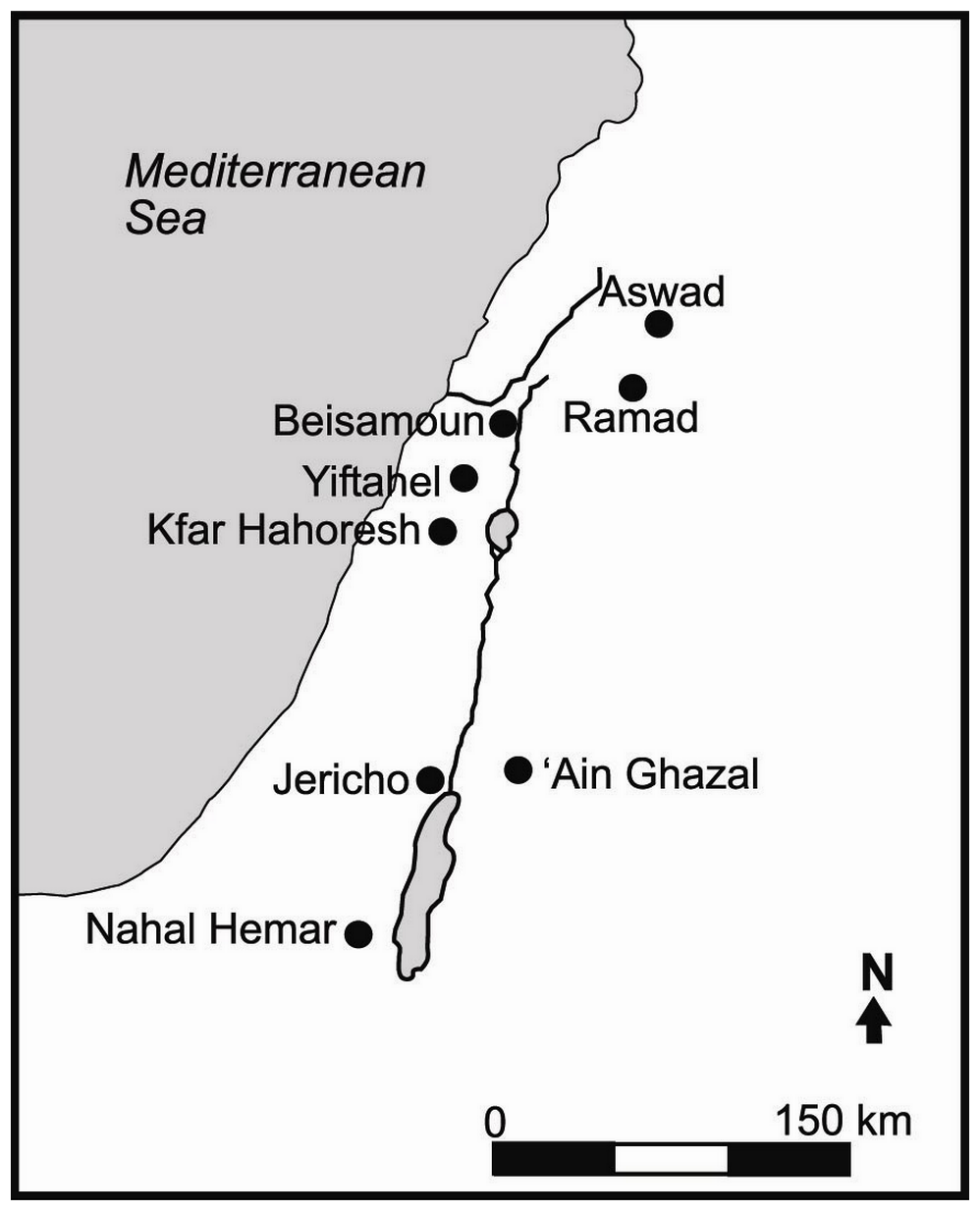
Map of the southern Levant. Archaeological sites where artificial remodeled skulls have been found are marked with black dots.

Modeled skulls have been found in archaeological sites from many parts of the world, as recently reviewed by Aufderheide [5] and Croucher [6]. In the Levant, plastered and remodeled skulls have been found in several PPNB sites, such as Jericho, Tell Ramad, Beisamoun, Kfar Hahoresh, Tell-Aswad, 'Ain Ghazal, and Nahal Hemar (Fig. 3) and are thus considered part of a mortuary practice typical of the PPNB [7]–[19]. This practice seems to have continued in Anatolia, as plastered skulls have been found at Köşk Höyük [20]–[21] and Çatal Hüyük [22] in much later PN contexts. It is still unclear how the central Anatolian plastered skulls relate to those of the Levant, when there are none of such plastered skulls in Anatolia during the PPNB, and none in the Levant during the PN.

For our case, it is important to note that the PPNB is characterized by the shift from hunter-gatherer groups to agricultural societies. This process took place on many different levels, e.g., demographic, societal, religious, economic and epidemiological levels [23]–[25].

The aim of this study is to examine the three Yiftahel plastered skulls from both anatomical and technical aspects, and compare them to other remodeled skulls from the Levant. This will be carried out in order to shed additional light on an intriguing mortuary practice during the advent of agriculture, thus catching a glimpse at an ancient society in the midst of a cultural and social revolution.

## Materials and Methods

### Archaeological specimens

The plastered skulls from Yiftahel (H1, H2 and H3) are housed in the Anthropological Collection at Tel-Aviv University, Israel. The excavation of the site of Yiftahel was authorized by the Israel Antiquities Authority (permit number 5252/2007). All necessary permits were obtained for the described study, which complied with all relevant regulations.

### Site location and primary condition

**Figure 4:**
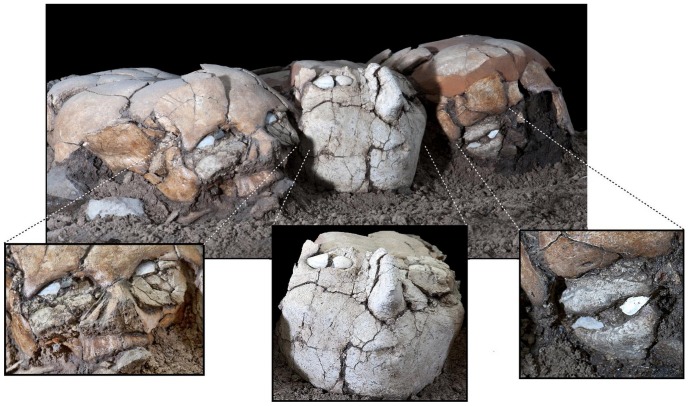
The three plastered skulls, following reconstruction and preservation processes. Details of each plastered mask are shown in the enlargements at the bottom of the figure. Homo 1 – left; Homo 2 – center; Homo 3 – right.

Three plastered skulls (Figs. 2,4) were found during the 2008 excavation season at the site of Yiftahel, in Area I. They were found buried together (L4187, Open area 402 near Building 501, see Figs. 1,2) in a row, facing west. They were dubbed Homo 1–3, from north to south. Furthermore, numerous burials were found under the floors or in pits, mainly in Area I, some of them with removed skulls [2]–[3].

All skulls were badly damaged, as a consequence of pressure from the surrounding sediment through the many years since their burial. Additional damage was caused during the excavation, when the upper part of the calvarium of Homo 3 was accidentally shattered.

### Preservation and removal from site

At the excavation site, the three skulls, along with the sediment around and beneath them, were wrapped in a plastic cover and covered in polyurethane foam, a diphenylmethane-4,4′-diisocyanate polymer which hardens upon contact with air.

### Restoration

In the laboratory, excess sediment was removed from around the three skulls, revealing the lower portions of the skulls and the full height of the mask on Homo 2. The bones and the plaster masks were cleaned of sediment residues using pure acetone, thus uncovering the shells embedded in the plastered eye of Homo 3.

Once cleaned, the bones were treated with glyptal 1276 5%, a lacquer cement used to strengthen them. Fragments of Homo 3's calvarium were reassembled. Sediment taken from the site, mixed with paraloid 25%, was used to fix the bone fragments onto the remains of the skull. The reconstruction was meant to match the elliptical form of the other two skulls.

The plaster in all three masks was treated with paraloid 10%, which served to reinforce it, as well as to fill minute cracks and avoid damage to the masks. Excess paraloid was removed using pure acetone.

The soil inside, around and beneath the skulls was injected with paraloid 25% to prevent collapse. Further support was achieved by spraying additional polyurethane foam around the sediment.

In all three skulls, areas from which bone fragments were missing were covered with wax to provide stability to the reconstruction. The wax was painted using oil paint, its color resembling the bones. The yellow color of the extra polyurethane around the skulls was covered using sediment mixed with paraloid 25%.

### Study

Computed tomography (CT) scans were performed using a Philips iCT-256 scanner (slice thickness 0.65 mm; voltage 140 kV; current 359 mA) at the Carmel Medical Center in Haifa, Israel. Cross-sections and volume rendering functions were utilized for detailed examination of the bones and plaster masks. Density of the plaster was determined using the circular region of interest (ROI) tool, upon virtual samples of 30.0 mm^2^ (except for the dental sockets, where samples were of 10.0 mm^2^), and expressed in Hounsfield units (HU).

Sex was determined using cranial criteria [26]. Age was evaluated using the degree of closure of ectocranial sutures [26]–[27].

## Results

### Homo 1

Although the glabella is quite smooth, the thickness of the supra-orbital margin and the large size of the mastoid processes (visible only in CT scans) suggest that Homo 1 may be a male. The anterior section of the sagittal suture was open ectocranially, the bregma was minimally closed, and the midcoronal suture was synostosed. This suggests that Homo 1 is an adult, most probably in the young (20–34 years) or middle (35–49 years) age cohort.

The frontal bone of H1 is broken into three distinct longitudinal fragments, the lateral ones slightly overriding the medial piece. Blood vessels engravings are noticeable on both sides of the frontal bone. The forehead appears smooth, with inconspicuous supraorbital ridges. Both supraorbital margins are mostly intact. The nasal bones are relatively well preserved (Fig. 4).

**Figure 5:**
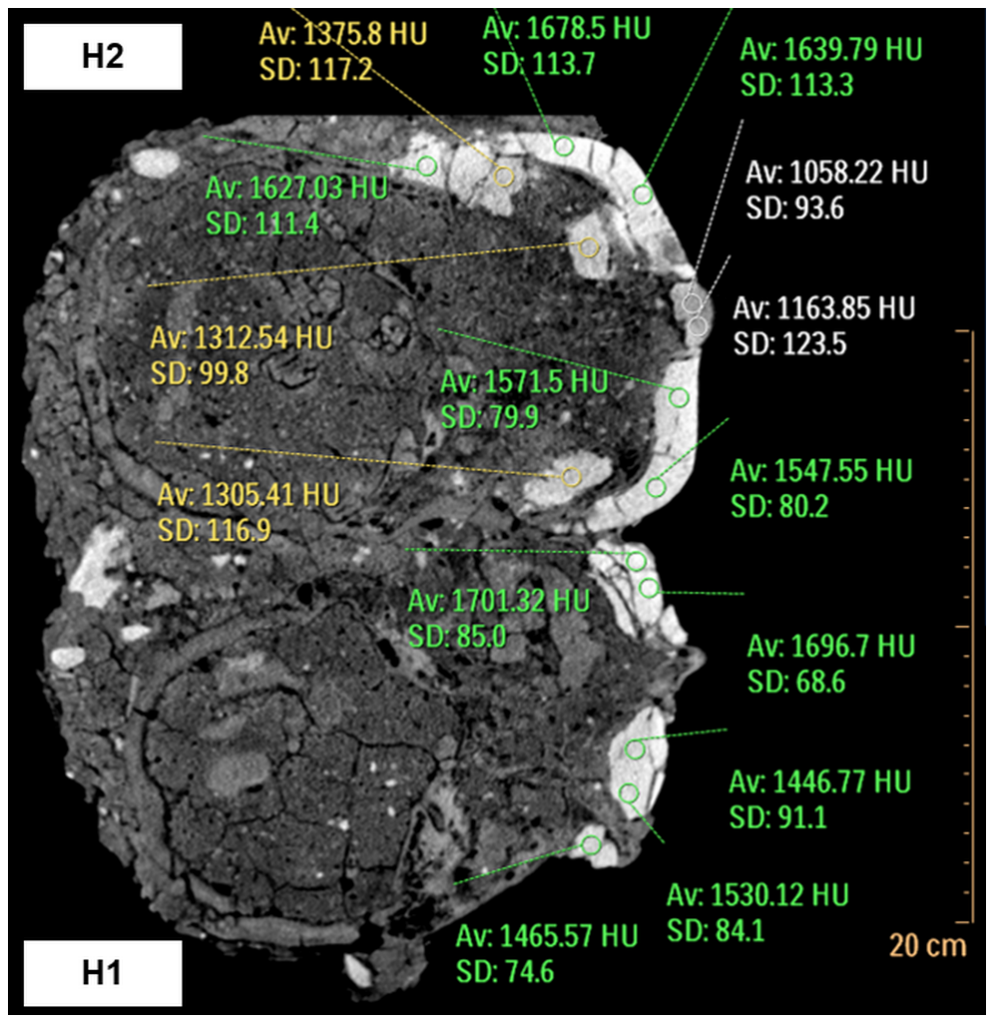
CT scan of Homo 1 and Homo 2. Scans of Homo 1 (lower half of figure) and Homo 2 (upper half of figure) showing results of the ROI analysis, indicating the density of the plastered masks. Densities below 1200HU are marked in white; between 1200–1400HU in yellow; and above 1400HU in green.

**Figure 6:**
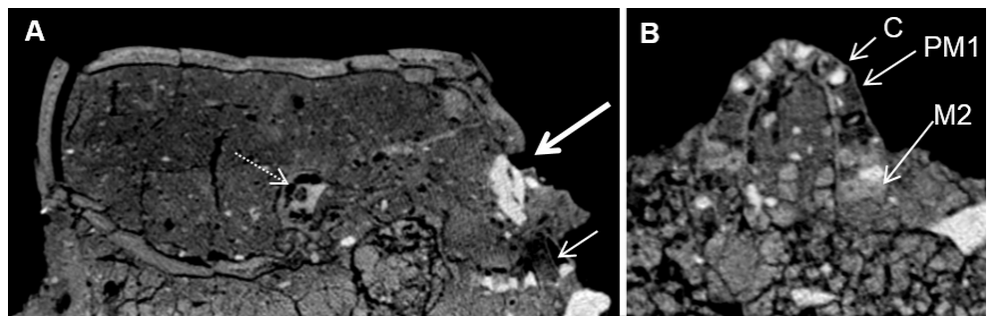
CT scan of Homo 1. In the sagittal section (A), note the orbital filling, in its correct anatomical position (thick arrow). The root of the upper right canine is missing (thin arrow). The apical part of the dental socket is empty, while the cervical part is filled with plaster. There is no mandible. The petrous bone is intact (dashed arrow). In the axial section at the level of the maxilla (B), note that the cervical part of the dental sockets for the incisors and the canines (C – right canine) are filled with plaster. The dental sockets of the right first premolar (PM1) and the left first and second premolars are filled with material of bone-like density. Most roots of the left first molar and the right first and second molars (M2) are present.

The orbital cavities are filled with plaster of comparable high densities, ranging from 1446.77 to 1616.42HU for the right socket, and from 1554.36 to 1701.32HU for the left (Fig. 5). On the right side, the filling presents a rectangular shape. Its upper part is tucked under the orbital rim, thus the eye of the plastered mask is placed in its correct anatomical position (Fig. 6). A portion of the plaster filling the orbit extends outward to cover the lateral orbital rim, the medial part covers the lacrimal bone, and the lower part overhangs the zygomatic process of the maxilla. A white shell fragment is placed above the plaster on the upper-right side of the filling, below the lateral region of the supraorbital margin. A black flint chip is placed medially to the shell, putatively representing the iris or the pupil.

The left orbital filling is more rounded in shape. A shell fragment, similar to the one on the right side, is placed on the upper right side of this filling. This arrangement gives the viewer the impression that the skull is looking diagonally, upward and to its right. No flint fragment was found in the left orbital filling. Instead, a plaster bead protrudes from just left of the shell. The left filling slightly covers the left nasal bone, leaving bare only the lower part of the nasomaxillary suture. This filling is cut off higher than the right filling, revealing a greater part of the zygomatic bone (Fig. 4).

The left zygomatic process of the frontal bone rests upon the mask of Homo 2, placed to the left of Homo1. The mandible and most upper teeth are missing, causing the body of the maxilla to be in contact with the sediment around it. Below and to the right of the maxilla, within the sediment, there is a thin elongated animal bone.

Regarding the maxillary incisors, canines and the right second premolar, CT images revealed that the apical areas of the dental sockets are empty, while the cervical parts are filled with plaster ranging from 1331.34 to 1645.72HU. The left premolars and the right first premolar appear to have been lost ante-mortem, since the alveolar bony sockets are obstructed by a material with bone-like density. There is a small amount of plaster on the cervical part of the socket of the right first premolar, but none in the area of the left premolars. The roots of the left first molar are present, from the area of the apex until the root trunk. The two buccal roots of the left second molar are visible as well. The area for the palatal part of the second molar and for the third molar is fragmented. No plaster was found on the region of the left molars. On the right side, the disto-buccal and palatal roots of the first and second molars are present from the apex to the root trunk. The mesio-buccal roots of both teeth are missing. Plaster fills their cervical part, but does not reach their apical part (Fig. 6).

Homo 1 can be examined from a lateral view only on its right side, as the left is obscured by the skull of Homo 2. Its right temporal foramen is filled with plaster. The material within the temporal foramen is slightly less dense (1391.61–1458.12HU) than the one inside the orbital cavities (Fig. 5).

The squamosal part of the temporal bone projects laterally. The external acoustic meatus and the mastoid process are not visible, being buried within the sediment. However, the petrous bones and the mastoid processes are identifiable in sagittal CT cross-sections (Fig. 6).

From a posterior view, only the upper part of the occipital bone is visible. CT analysis showed that the occipital bone is fragmented, but all the pieces seem to be present. The lambdoid sutures are split open, as the occipital and parietal bones protrude in opposite directions, the former upward, and the latter downward.

On the left parietal bone, approximately 2.5 cm lateral to the lambda, an area of discoloration (27 mm×15 mm) with rugged and uneven borders is visible. On the anterior third of the left lateral border, a slightly sunken area (4 mm×4 mm) is apparent. Three horizontal stripes criss-cross the discolored area, approximately 4 mm from one another. The two anterior stripes are connected on their left side to the sunken portion of the discoloration. The nature of the lesion could not be determined.

The top of the calvarium is crushed and sunken, causing the bregma to be the lowest point of the calvarium. The middle area of the frontal bone and the posterior area of the parietal bone are oriented vertically. The medial part of the coronal suture is fused, while the lateral section appears to have been ripped apart by the pressure applied on the skull from above. The sagittal suture is fused as well, but to a lesser extent.

### Homo 2

The somewhat protruding glabella and the large size of the mastoid processes may imply that Homo 2 was a male. Based on the significant closure of the anterior-sagittal and the bregma sutures, and the complete obliteration of the midcoronal suture, the skull belonged to an adult individual, probably within the middle age category (35–49 years), albeit a greater age (50+ years) could not be dismissed.

The entire facial region of the skull, up to the slightly prominent supraorbital ridges, is covered by a plaster mask depicting a human face (Figs. 2,4). The eyes are represented by two elongated white shell fragments, placed within shallow indentations in the plaster. In the left eye, a vertically oriented spiral shell is placed between the two shell pieces. This part is missing from the right eye, leaving a small empty space between them. The face depicted by the mask appears to be looking forward. The slender nose, lacking nostrils, is represented by a protruding piece of pyramid-shaped plaster. The plaster depicting the lowest part of the nose has a density ranging from 1058.22 to 1163.85HU, while the rest of the outer part of the mask has a density of 1474.25–1678.5HU (Fig. 5). The lower left part of the nose, where the alar fibrofatty tissue would normally be, is smoother than the rest of the mask, as its rugged plaster overcoat is missing. A small elliptical concavity underneath the nose represents an open mouth. The lateral areas at the level of the nose project sideways, as if representing prominent cheekbones. There are two vertical and elongated gashes on the right side of the mask. The first one, which is not continuous, is placed beneath the putative cheekbone. The other is near and under the mouth. It is hard to determine whether these are due to damage caused by the passage of time, or if they are intentional features, perhaps representing scars (Fig. 4).

**Figure 7:**
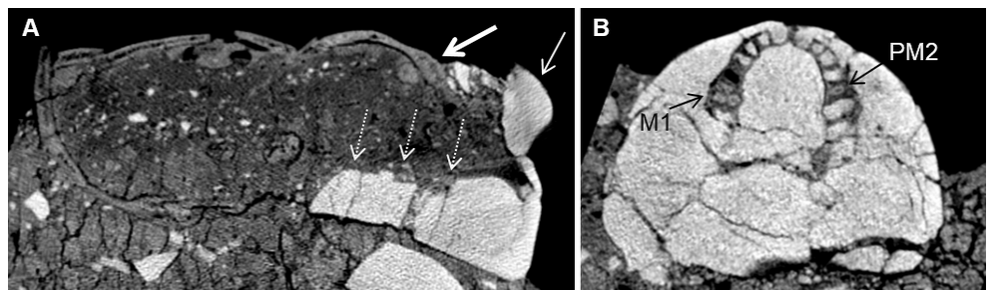
CT scan of Homo 2. In the sagittal section (A), note that the superior part of the plastered nose (thin arrow) ends at the level of the supraorbital ridge (thick arrow). There is therefore a discrepancy between the mask's features and the correct anatomical position. Note also the thick layer of plaster (dashed arrows) at the base of the skull, instead of the missing mandible. In axial section at the level of the maxilla (B), note that the cervical part of the dental sockets for most teeth are filled with plaster. The roots of the left first molar (M1) and the right first premolar (PM2) are present.

The CT scans enabled us to see that the mandible had been removed prior to the fitting of the plaster mask, causing a discrepancy between the mask's features and their anatomical position within the skull. A thick layer of plaster (up to 4 cm in height) runs underneath the skull anterior to the petrous bones, encasing the maxilla while filling the palate, and, at its most anterior part, constituting the chin of the mask (Fig. 7).

Regarding most of the maxillary teeth, the apical areas of the dental sockets are empty, while the cervical parts are filled with plaster (density of 1116.28–1484.83HU). Plaster also partially fills the apical part of the dental socket of the left lateral incisor. The right second premolar is broken at its cervical part, and the crown is missing. The root canal is visible throughout most of the root. The roots of the left first molar are also present, from the area of the apex to the root trunk. Plaster covers the cervical parts of the roots in both teeth (Fig. 7). The plaster that covers the cervical portions of all maxillary dental sockets is continuous with the thick layer of plaster serving as a base or chin for the mask.

The temporal foramina are filled with plaster having a density of 1219.34–1371.44HU (Fig. 5). On each side, the filling of the temporal foramen is continuous on its lowest part with the thick layer of plaster constituting the chin of the mask and covering the inferior part of the skull until the level of the external auditory meatuses.

The orbital cavities are filled with plaster in their anterior part (density varying between 1312.54 and 1645.19HU), while the posterior part is filled with a soil-like material (Fig. 5). The eyes depicted on the mask are not in correct anatomical position, as their most superior areas rest on the skull's supra-orbital ridges. Likewise, the nose of the mask, made of a solid piece added to a void in the mask, runs from the level of the supra-orbital ridges (Fig. 7) to the level of the orbital surface and zygomatic arch. The nasal cavity itself is filled with soil.

The superior part of the skull presents a wavy surface, most probably caused by unevenly distributed pressure from the overlying soil. Two areas on the antero-lateral aspects of the parietal bones are missing. Toward the back of the skull, CT analysis revealed a hollow area, lacking sediment infilling. The posterior extremity of the sagittal suture has been pried open by the upward protrusion of the parietal bone fragments adjacent to it. The occipital bone is entirely hidden within the sediment.

### Homo 3

The thinness of the supra-orbital margin, the smoothness of the glabella and the small size of the mastoid process suggest that Homo 3 is a female. Only the anterior lateral suture could be used to evaluate age. Its minimal closure is more likely to be found in young (20–34 years) to middle adults (35–49 years).

**Figure 8:**
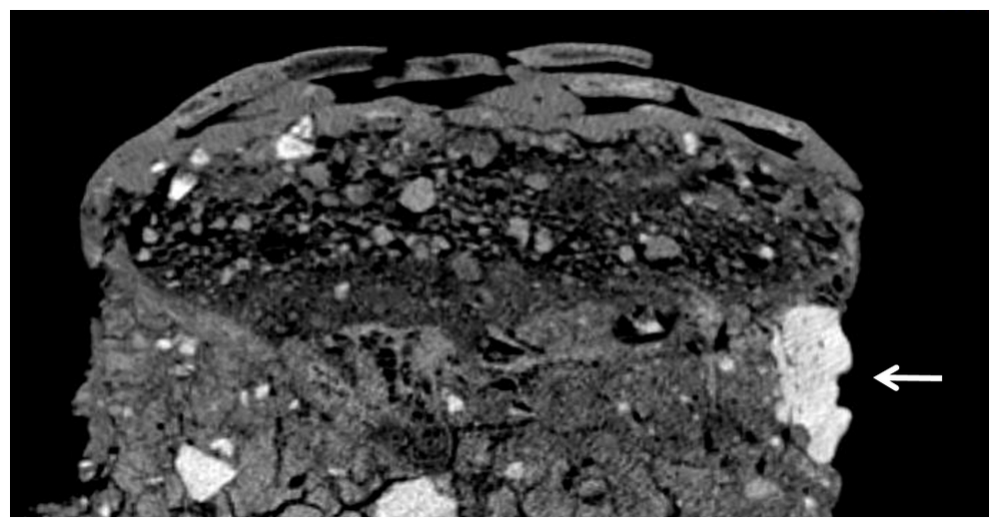
Sagittal cross-section of Homo 3. The cross-sections shows the plastered eye (thick arrow) in its correct anatomical position, within the right orbital cavity.

Homo 3 was the most damaged of the three skulls (Fig. 2). The refitted skull lacks many parts, including large areas of the frontal and parietal bones. On the left side of the skull, only the superior orbital rim of the left eye and a fragment of the nasal bone remain. The right orbital cavity is sealed with an eye-shaped plaster filling (Figs. 4, 8). Its density (1477.89–1604.85HU) resembles the material in the orbital cavities of Homo 1 and the mask of Homo 2. The rounded plaster filling is composed of two semi-lunar pieces, one above the other, resembling thick eyelids. On the lateral areas of the gap between them, two pieces of white shells, similar to the ones in the mask of Homo 1, possibly represent the sclera. In the middle of the gap, inferior to the supraorbital notch, a protruding plaster bead putatively embodies the iris or the pupil. The mask appears to be looking downward, possibly due to the tilting of the entire skull toward Homo 2. The left supraorbital margin and zygomatic bone currently stand empty (Figs. 2,4), but one might hypothesize that a similar plaster filling was once housed in the right orbital cavity as well.

**Figure 9:**
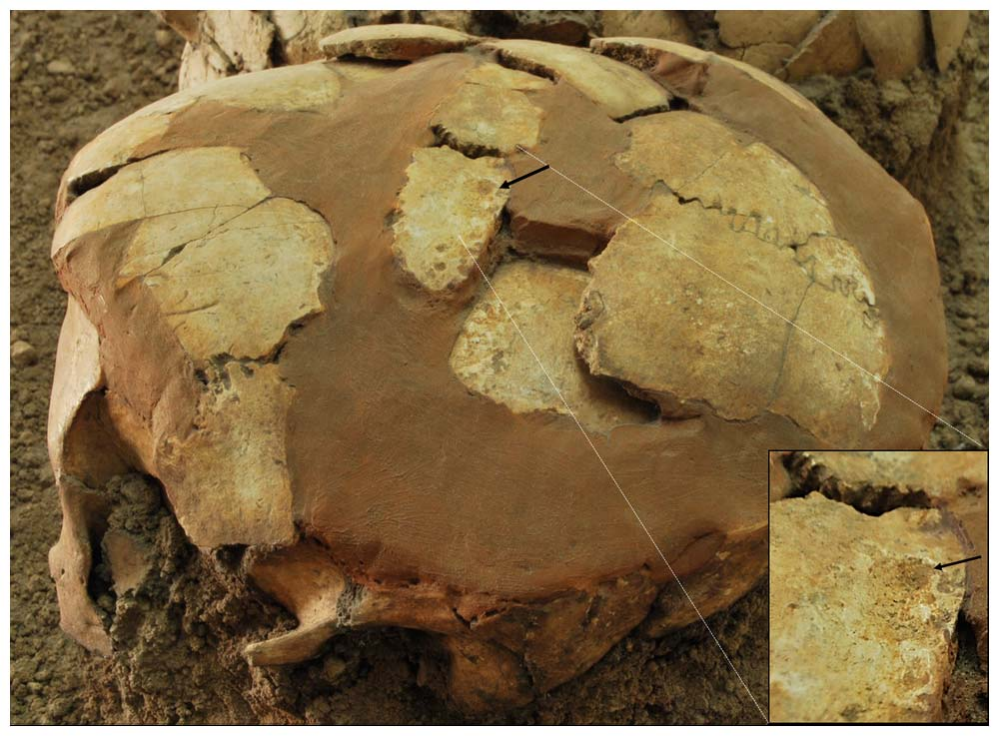
Oval depressed ante-mortem skull trauma in Homo 3. The trauma is noted by a black arrow. Enlargement of the trauma is shown at the bottom right part of the figure.

On the left parietal bone, about 7 mm lateral to the anterior part of the sagittal suture, a small, oval (11.6×6.8×1.8 mm) healed (ante-mortem) depressed fracture is present (Fig. 9). The left zygomatic arch is missing, but the external acoustic meatus and part of the mastoid process remain above the sediment. A small part of the superior area of the occipital bone can be seen at the back of the skull.

## Discussion

### The Yiftahel plastered skulls and comparisons with other specimens

In the Levant, planned burial customs, performed for ritualistic purposes, date as far back as the Middle Paleolithic [28]. Skull-related funerary practices began during the late and the final Natufian of the southern Levant (10,500–9,800 BCE), as evidenced in the Hayonim Cave and Eynan sites, where skulls were detached from the rest of the skeleton [14]. In PPNA Jericho, group burials of isolated unadorned skulls (nested skulls), organized in various configurations, have been found [11]. The same phenomenon was reported in the EPPNB site of Motza [29].

During the Mid-late PPNB, mortuary customs developed, involving the artificial remodeling of skulls, evidence of which has been found across the Near East. Skulls covered by plaster masks have been located in Jericho [7], [11], Beisamoun [10], 'Ain Ghazal [13], [15], Kfar Hahoresh [16], Tell Ramad [8]–[9], and Tell Aswad [19]. Three plastered facial masks, without the human skulls, were found buried together in a pit at 'Ain Ghazal [30]. At Nahal Hemar, three skulls ornamented with asphalt motifs and one burned skull were found [12], [31]. In Ujrat el Mehed (southern Sinai), adult skulls were removed from the post-cranial skeleton, similarly to other Neolithic Levantine sites [32]. Other special remains include a skull painted with red ochre from Tell Abu Hureyra [33], a skull decorated with red and black substances from 'Ain Ghazal [34] and painted stone masks from Nahal Hemar [35] and the area of Er-Ram (exact provenance unknown) [36]. Thus, the three plastered skulls uncovered at Yiftahel join a growing record of artificially treated skulls from the Levantine PPNB.

Most of the plastered skulls found to date belong to adult individuals, and both sexes are represented. All three plastered skulls from Yiftahel very likely belong to adult individuals. Although very few criteria could be used to evaluate age, it seems that Homo 2 is slightly older than Homo 1 and 3. Homo 1 and 2 seem to have been males, and Homo 3 a female. It should be noted that determination of sex and age based solely on visible traits in the skull is problematic, due to the range of ages that can be indicated by any given character, the overlap of identifiable traits between males and females, and the effect of ancestry on these traits [37]. This caveat is especially true in the case of the plastered skulls, as some of the traits (e.g., sphenotemporal suture, nuchal crest or mental eminence) are not available for study.

The healed blunt trauma to the head of Homo 3 may have occurred ante- mortem accidently or following a violent encounter [37]. Based on the size and location of the healed injury, it is unlikely to have caused lasting damage to Homo 3. The prevalence of skull trauma has been estimated at 2.9% during the Neolithic period. It was more common in the earlier Natufian period (16.7%) [38] and in the later Chalcolithic, Bronze and Iron periods (26.67%) [39].

Cultural and social ties between sites in the Levant may be inferred from the resemblance in their funerary practices. It would seem that during the PPNB, the Levantine sites were characterized by an overall similar material culture, although specific localized variations can be identified [24], [40]–[41]. The anthropomorphic plastered mask covering the facial area of Homo 2 closely resembles those found covering skulls in Jericho [11] and Kfar-Hahoresh [18], as well as the three masks from 'Ain Ghazal [30]. The mandibles of the three plastered skulls from Yiftahel had been removed, similar to those of Kfar Hahoresh [17] and 'Ain Ghazal [13], [30], but unlike skulls from Jericho [11] and Beisamoun [10]. The lack of teeth in the plastered skulls from Yiftahel may be due to dental evulsion prior to modeling, as has been suggested regarding other modeled skulls from the Levant [9]–[12]. The fact that the top of the calvarium has remained uncovered with plaster is rather common, with the exception of the unique asphalt braided patterns on the skulls from Nahal Hemar [12]. Based on the Nahal Hemar specimens, it has been suggested that a headdress could have once covered the bare upper areas of the plastered skulls [24]. The use of shells to represent the eyes has previously been noted in plastered skulls from Jericho [11] and Beisamoun [10]; but the black flint used to depict the pupil of Homo 1 is, as far as we know, insofar one of its kind.

The partial orbital masks of Homo 1 and 3 are quite different from the facial masks found in other PPNB sites. The filling with plaster of the temporal foramen and the lower part of the alveolar cavities in Homo 1, which is reminiscent of the mask of Homo 2, may indicate that the mask of Homo 1 is actually the remnant of a once more complete plaster mask. In any case, differences in style are evident between the three masks, and Homo 1 and 3 are similar in that the eyes of the masks are in their correct anatomical position. The fact that the two similar masks bracket the one different wide-faced mask brings forth a sense of symmetry or harmony, which may reflect both spiritual and aesthetical purposes, and thus could have been intentional. This rare find can be hypothesized to hold a discrete meaning, such as the representation of two distinct classes of people, as was suggested previously [3]. Moreover, in this case, the fact that Homo 1 and Homo 3 are putatively younger than Homo 2 may have had an impact on the choice of mask type, as well as on their positioning on both sides of Homo 2.

Many of the masks found to date, including Homo 2, present a broad facial type. It has been suggested that cranial morphology was a criterion for selecting skulls to remodel, and that when appropriate skulls could not been found, *in vivo* or post-mortem deformations were undertaken, in order to create the required illusion of a broad and wide cranial dimensions [12]. The removal of the mandible has also been explained as a means to create a flat surface, on which the skull could rest when placed on shelves [11]. CT scans of Homo 2 showed that a thick layer of plaster was placed instead of the mandible, creating an artificial chin. In the case of the plastered skull from Kfar Hahoresh, it was deduced that this layer of player, compensating for the lack of mandible, serves as a platform or base for the mask [18]. The same logic could be applied here, regarding the thick layer of plaster beneath the skulls of Homo 2, which may have been served to increase the stability of the mask. Similarly, the filling of the temporal foramina and of the anterior parts of the orbital cavities in Homo 1 and 2, reminiscent of the mask of Homo 1 in Kfar Hahoresh, could be interpreted as a way to anchor the plaster mask unto the skull.

The burial of the three plastered skulls together echoes the eight plastered skulls from Tell Ramad [14], the seven skulls from Jericho [7], the two plastered skulls from Beisamoun [10], and the three masks found buried together at 'Ain Ghazal [30]. In the case of Yiftahel, any putative meaning of the co-burial in a single pit has been preserved, as it was decided not to separate the skulls during the restoration process.

The discrepancy between the features depicted in the mask of Homo 2 and the anatomical position of the corresponding organs is reminiscent of that noted in the masks of Kfar Hahoresh [18] and 'Ain Ghazal [30]. In both Homo 2 from Yiftahel and Homo 1 of Kfar Hahoresh, the orbital cavities were filled with plaster, and the eyes were depicted at the level of the frontal bone. Unlike the specimen from Kfar Hahoresh, in the case of Homo 2, the position of the mouth corresponds with the level of the maxilla. The nose seems to be pushed upward, as its base is in line with the lowest part of the orbitae. In contrast, the plaster eyes of Homo 1 and Homo 3 are in their proper anatomical positions.

### Reconstruction of the modeling technique

The remodeling technique used to create the mask of Homo 1 from Kfar Hahoresh was analyzed and described by Hershkovitz *et al.* [17]–[18]. In that specimen, at least four distinct layers of plaster could be determined, each used for a different stage in the remodeling process. The differences in radio-density were shown to result from the technique used to create the material, i.e. the proportion between the ingredients and the amount of calcination. Chemical analyses of two of the Jericho modeled skulls showed dissimilarities in the product used to create them. In one skull (1955-565), a mixture of two types of material was utilized. Interestingly, the analysis of the second skull (JPE 121.32) revealed that one homogenous material was used, contrasting with the visual appearance of two layers [42]. A recent analysis of the plastered floors from Area I of Yiftahel showed that all samples were of lime plaster exposed to high temperature [4]. In the case of Homo 2, minute distinctions based on the CT scans could not be evaluated, as densities of the sediment, bone and plaster were relatively similar. ROI analyses enabled a distinction between two types of plasters. The majority of the plaster in all three masks was quite dense. Less dense material was used to fill the temporal foramina and some of the dental sockets in Homo 1 and 2, and to create the nose in Homo 2. However, as chemical analyses have not been performed regarding the plaster of the masks, it is hard to determine whether these findings represent true differences in the material used.

**Figure 10:**
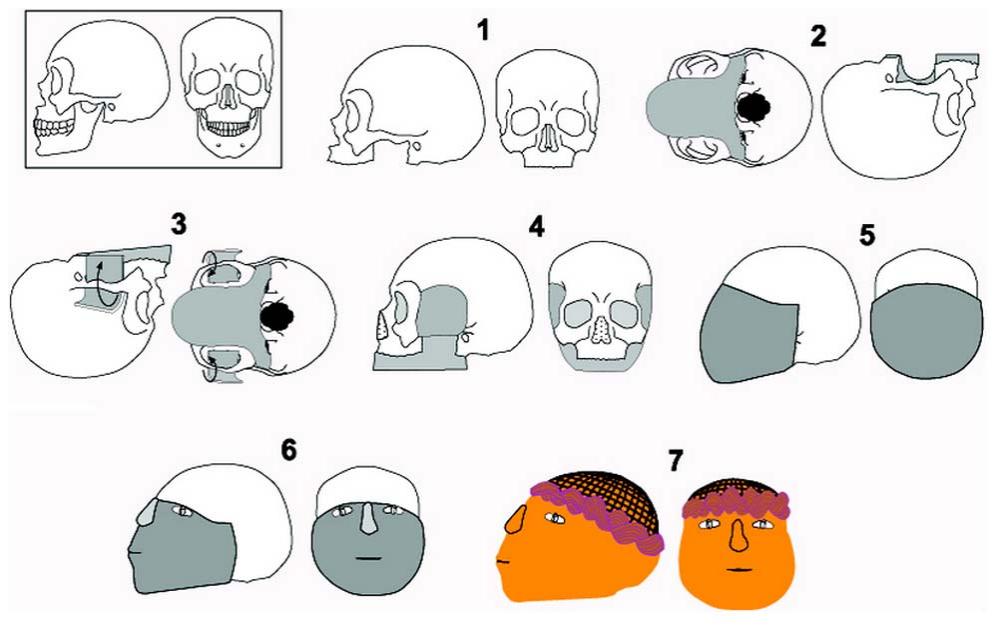
An artists reconstruction of the process of modeling Homo 2s mask. Phase 1: Removal of the mandible and upper teeth. Phase 2: Preparation of the base of the mask. Phase 3: Filling of the temporal foramina with plaster. Phase 4: Filling of orbital and nasal cavities. Phase 5: Positioning of the plaster mask on the facial area, while anchoring it on the plaster base created in phase 3. The upper part of the mask is at the level of the supra-cilliary eminence, thus the superior part of the calvarium is not covered. Phase 6: Modeling of the mask by adding shells to create the eyes (at the level of the frontal bone), adding a plaster nose (its base at the level of the inferior part of the orbitae), and tracing the mouth (at the level of the maxilla). Phase 7: Decoration of the mask by adding color and/or natural materials to mimic a headdress.

Based on the analysis of the CT scans and a comparison with previous studies on other plastered skulls [12], [17]–[18], [41]–[42, we attempted to reconstruct the phases necessary to fashion a plastered skull such as Homo 2. An artists reconstruction of the phases is presented in Figure 10. Firstly, the mandible and upper teeth were removed. Secondly, the base of the mask was prepared, covering the inferior part of the skull until the level of the mastoid processes, and then the temporal foramina were filled with plaster. Afterwards, the orbital and nasal cavities were filled with a soil-like material. A plaster mask was then anchored upon the plaster base, covering the facial area up to the level of the supra-ciliary eminence while leaving the superior part of the calvarium uncovered. Subsequently, the facial features were modeled upon the mask, by using shells to create the eyes (at the level of the frontal bone), adding a plaster nose (its base at the level of the inferior part of the orbitae), and tracing the mouth (at the level of the maxilla). Lastly, the mask was putatively decorated by adding color or and/or natural materials to mimic a headdress.

### Suggested interpretations of plastered skulls

A recent review of overmodeled skulls from around the world supplied a summary of interpretations for this practice, among different groups and in different periods. Both religious and secular interpretations were provided. Modeled skulls were used, among others, as a memorial for a deceased individual, in rituals of necromancy as an intermediary between the living and the dead, as a protection against enemy spirits, or as a relic in praise of past victories [5].

The plastered skulls from the Near East are most commonly explained as part of an ancestral cult [6]–[7], [10]–[14], [23], [30], [40], [43]. The social shift from mobile hunter-gatherer groups to a sedentary life in agricultural settlements could have produced a need to mark territories, and thus to prove ones association with a certain location. The worship of earlier generations, possibly through ceremonial display of their plastered skulls, may have been part of an effort to plant roots and establish “ancestral property rights” to a piece of land.

Artificial deformations and dental evulsion, when performed on relatively young individuals, may reflect an attempt to render a more elderly look, suited for rituals of ancestor cult [12]. It should be noted, however, that the claim that post-mortem dental evulsion was a common practice has been contested, based on the possibility of tooth loss from natural causes or from damage to the modeled skulls, and based on the finding of teeth in several specimens [44]. In the case of the Yiftahel skulls, the fact that most dental sockets are empty (of either bone or plaster) implies that tooth loss was not ante-mortem in most cases. The presence of several partial dental roots, however, may indicate an only partially successful attempt to forcefully remove the teeth post-mortem, resulting in breakage of the crowns and the remaining of the roots within the dental sockets.

The interpretation of ancestral cult has been contested [45] in light of the variety of individuals, of both sexes and different ages, whose skulls underwent plastering. Perhaps the idea of ancestral worship should be expanded from the traditional view of ancestors solely as old men, to encompass any influential person from the past (men, women and even children) with familial ties to the then present settlers of the territory.

A second theory, based on observations of modern tribal societies, states that the skulls could have been those of vanquished enemies, safeguarded as trophies of war to prove ownership on a land [14]. This interpretation, among others, has been suggested by Kenyon [7] regarding the plastered skulls of Jericho. In the case of Yiftahel, as there are several local burials with cranial removals at the site, it is difficult to accept this explanation in our case.

A third explanation suggests that plastered skulls are part of a mythological, spiritual, or religious context [3], [13], [20], [24], [46], perhaps related to a symbolic reincarnation or to the re-introduction of the dead to the everyday life of the living. Specifically, the use of plaster in connection with several mortuary practices has been viewed as both connecting and separating the world of the living from that of the dead [24]. It has also been suggested that plastered skulls could have been used in various ceremonies, as a protection from evil or in connection with fertility, fecundity and “life-force” rituals [20], [47]. Headless statues and plastered figurines resembling faces portrayed in the masks of the skulls have also been found in several sites [14]. This may suggest that common religious beliefs and practices were spread throughout the Levant during the PPNB.

Fourthly, it has been proposed that the skulls were modeled as homage to the dead [7], [13], [20]. The fact that modeling of skulls seems to have been reserved for specific individuals may be interpreted in connection with growing social complexity, with the need to emphasize differential status, either inherited or due to special actions during life (cult of “heroes”) [8], [24], [48]. This interpretation is compatible with the representation of both sexes and all ages in modeled skulls. In the context of celebrating the dead, there is a debate in the literature as to whether the masks portray specific persons, or rather represent general iconic human features [11]. It seem to us that the paucity of recognizable facial characteristics (except for the putative scars), as well as the disproportion of Homo 2s facial features, fit better the latter theory. The incompleteness of the masks of Homo 1 and Homo 3 allude to general, rather than specific, portrayals as well. Nevertheless, it should be taken under consideration that additional details pointing to a particular individual (such as hairstyle or ornaments) could have been made of degradable materials, and therefore be missing from our examination. This debate, however, may be moot, as the passing of time between the first burial to the use of plastered skulls in rituals may have bridged the recollection of a specific person by a closely knit group to the more general, depersonalized and inter-generational memory connecting a larger community [46].

Lastly, the skulls may have been remodeled in the framework of what we designate as artistic expressions. An artistic purpose for plastered skulls in the Levantine PPNB has rarely been alluded to previously [49], however it has been suggested regarding over-modeled skulls from other provenances in the world [5]. The knowledge of the raw materials properties and the knowledge of plastering by artisans, as expressed in several Levantine skulls [41], may be part of a system of craft specialization of the Neolithic communities, although in a non-canonized technical production in which some plastered skulls depict high skills and others show less dexterity. Community craft specialization is one of many patterns proposed for specialized production by Costin [50]–[51], based on the combination of her four parameters which could be the case of independent, nucleated, kin-based, probably part-time artisans (and see [52]). As the creation of the plastered skulls would have demanded time and craftiness, it seems to us that their aesthetical aspect, whether primarily artistic, or the secondary outcome of a ritualistic concept, cannot be denied; and those involved in these activities should be designated as probable part-time specialists.

## Conclusions

Anatomical and technical study of the three plastered skulls from Yiftahel allows us to position them within the record of artificially remodeled skulls from the Levant. The similarities and differences in style and technique of remodeling may be used to shed light on the PPNB populations, in terms of social, economic and/or cultural exchanges. The shift from hunter-gatherer groups to agricultural settlements during the PPNB entailed many life-style changes, including the increasing need to mark territories, probably accompanied by variations in spiritual and religious beliefs, as reflected in mortuary practices.

In the case of Yiftahel, the differences in modeling between the skulls and their arrangement may suggest a social hierarchy within the Yiftahel community, probably based on age and sex. Future studies on their relation with other burials at the site using ancient DNA methods may shed further light on the plastered skulls phenomenon, which remains a fascinating subject for debate.
